# From Genes to Ambiguity: A Case Study Exploring the Enigmatic Connection Between Chromosome 13q Deletion Syndrome and Ambiguous Genitalia

**DOI:** 10.7759/cureus.45168

**Published:** 2023-09-13

**Authors:** McKenna Yost, Terry Johnson, Jacob Kaiser, Connor Yost

**Affiliations:** 1 Obstetrics and Gynecology, A.T. Still University School of Osteopathic Medicine in Arizona, Mesa, USA; 2 Pediatrics and Child Health, Pediatric Associates, Wichita Falls, USA; 3 Internal Medicine, A.T. Still University School of Osteopathic Medicine in Arizona, Mesa, USA

**Keywords:** chromosome deletion, 13q chromosome, cytogenetic disorders, cytogenetic analysis, urogenital abnormalities

## Abstract

During development, the deletion of DNA from chromosome 13's short arm (q) causes a chromosomal abnormality known as chromosome 13q deletion syndrome. Chromosome 13 terminal deletions are rare and may cause various congenital disabilities, and only a few cases have been reported in the literature. The extent of chromosome 13q deletion syndrome changes lacks consistent clinical features, with no recorded cases of genital ambiguity until now. We report the case of a newborn male patient whose testes had descended on both sides; he had ambiguous genitalia, and the dorsal surface of his penis was attached to his scrotal sac. An abnormal karyotype (46, XY, deletion (13) q33) was discovered by using a G-banding analysis of chromosomes in a blood sample taken from the periphery, which revealed a deletion of chromosome 13 at the end of the first 10 cells. We can better characterize chromosome 13q deletions by establishing stronger correlations between karyotype and the distinctive phenotypes of haploinsufficient genes found on the chromosome.

## Introduction

Human cytogenetic disorders are caused by rearrangements of DNA that lead to the acquisition, deletion, or disruption of genes, which are dosage-sensitive [1]. Understanding the relationship between genotype and phenotype is crucial for diagnosis, prognosis, and genetic counseling. Hence, it is important to investigate chromosomal rearrangements with similar genomic imbalances [2].

Chromosome 13q deletion is a chromosomal defect that occurs when genetic information is removed from the short arm (q) of chromosome 13. This syndrome was first defined in the 1960s, and it has since been associated with a wide range of birth defects caused by deletions near the end of chromosome 13 [3]. The clinical manifestations of this condition vary greatly, and researchers have attempted to establish a link between genetics and phenotype [4]. The size of the deletion and the degree of mosaicism determine the phenotypic outcome [5]. Prominent clinical indications include brain, eye, heart, and kidney malformations associated with mental disability and developmental restrictions [6]. Other manifestations include bicornuate uterus, imperforate anus, imperforate anus with vaginal cloaca or fistula, hypospadias, and penoscrotal transposition [4,7]. The ephrin B2 (EFNB2) encoding gene, located in the 13q33.3-q34 region, and the endothelin receptor type B (ERTB) encoding gene, located in the 13q22.1-31.3 region, are potential candidate genes for reported anorectal/urogenital malformations [5]. Moreover, male neonates with ambiguous genitalia are more likely to have intellectual disability, retinoblastoma, and abnormalities of the genitourinary and digestive systems if the 13q chromosome is deleted [8].

Despite several attempts to correlate 13q deletions with specific symptoms, researchers have yet to establish a definitive relationship between 13q alterations and phenotypes [2]. However, genotype-phenotype analysis has been employed to identify the genetic locus(es) responsible for various disorders [9]. In this report, we present a rare case of chromosome 13q deletion syndrome resulting in ambiguous genitalia.

## Case presentation

A newborn male weighing 1830 grams, born via spontaneous vaginal delivery at a gestational age of 37 weeks, was brought to the nursery due to a lack of interest in feeding. The patient's initial complete blood count (CBC) revealed a platelet count of less than 10 x 10^9^/L, with a white blood cell (WBC) count of 3.2 x 10^9^/L. His mother had a history of delivering low-birth-weight babies, but none weighing less than 2500 grams.

**Figure 1:**
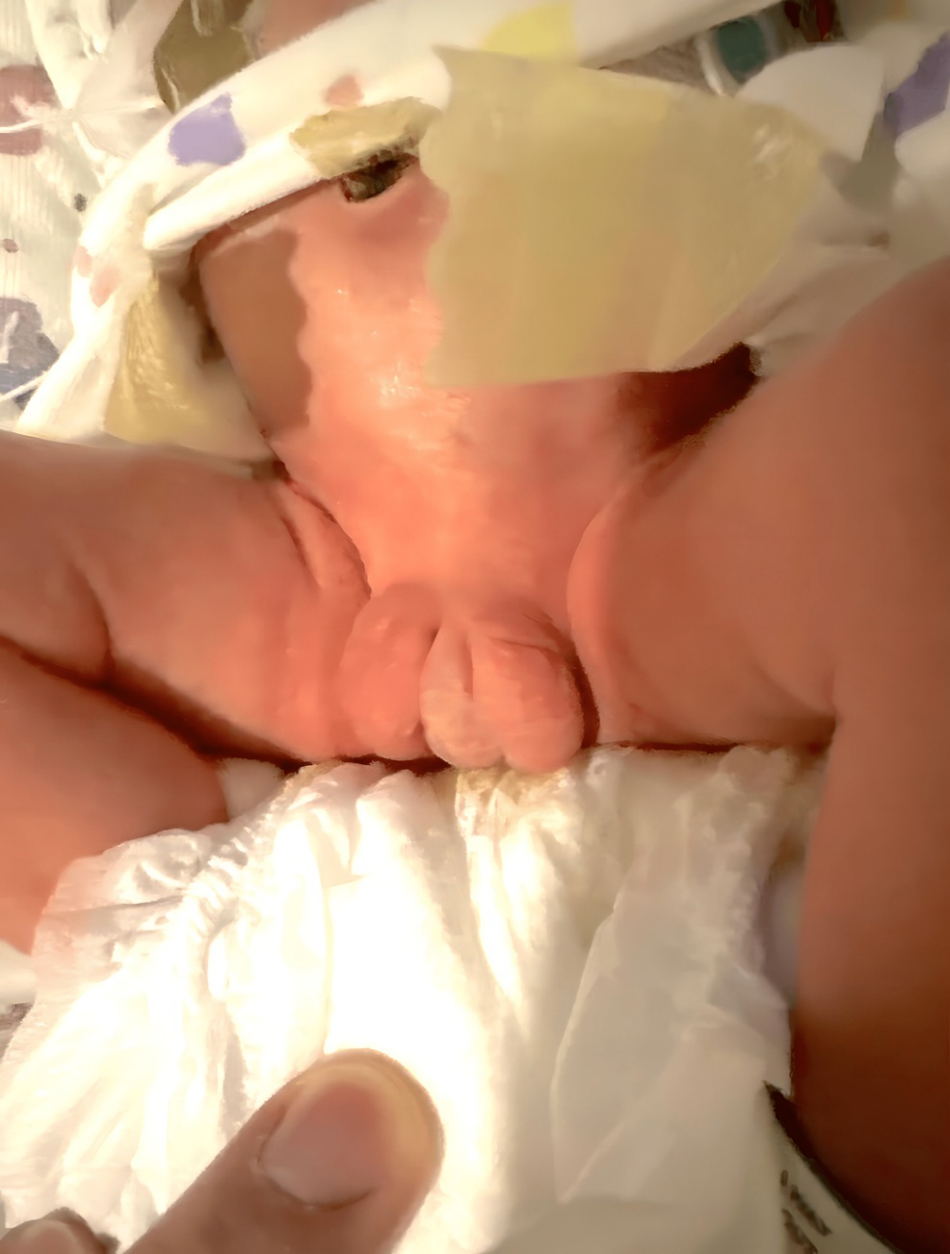
Ambiguous genitalia

Initially, a comprehensive physical examination was performed, assessing all vital signs, including the head, eyes, nose, and throat (HENT), cardiovascular system, pulmonary system, abdominal region, musculoskeletal system, skin, and neurological system. No abnormalities were found in these areas. However, the examination of the genitourinary organs revealed that the testes had descended bilaterally. The genitalia appeared ambiguous and the dorsal surface of the penis was found to be adhered to the scrotal sac (Figure 1).

Chromosome analysis

Due to the presentation of ambiguous genitalia, a chromosome analysis with the G-banding method was performed, and the patient's karyotype was found to be abnormal (46, XY, deletion (13) (q33)), which revealed a loss of chromosome 13 in the first 10 cells. Intellectual impairment, developmental delay, craniofacial dysmorphism, microcephaly, epilepsy, hypotonia, skeletal deformities, short stature, and cardiac and renal malformations are among the symptoms associated with terminal deletion of 13q. With the exception of the patient's genitalia, no other congenital abnormalities were detected.

Diagnosis

After the physical examination, the patient was diagnosed with newborn bacteremia, small for gestational age (SGA), respiratory distress syndrome (RDS), thrombocytopenia, decreased activity tolerance, and chromosome 13q deletion syndrome. The patient was admitted to the neonatal ICU (NICU). Various laboratory tests were performed and most results were as expected. The abnormal laboratory test results are presented in Table 1.

**Table 1:** Lab values during admission to NICU NICU: neonatal intensive care unit; WBC: white blood cells; MCH: mean corpuscular hemoglobin; RDW: red blood cell distribution width; NRBC: nucleated red blood cells; AST: aspartate aminotransferase

Lab test	Patient value	Normal range
WBC	3.2	9-34 K/CMM
MCH	39.1	24-34 PG
RDW	18.6	11.5-14.5%
Platelets	<10	150-400 K/CMM
Adjusted WBC	3.2	9-34 K/CMM
Lymphocytes	66	21-41%
Monocytes	1	2-10%
Manual NRBC per 100 cells	34	<=0%
NRBC, absolute	1.09	<=0K/CMM
Absolute neutrophil count	1.06	2-7.50 10^3^/UL
Segs absolute	1.02	6-26 K/CMM
Monocytes absolute	0.03	0.40-3 K/CMM
Macrocytes	Present	
Polychromasia	1+	
Target cells	Present	
Reactive lymphocytes	Present	
Comprehensive metabolic panel test		
CO_2_	28	20-24 MEQ/L
Total protein	4.5	6.1-7.9 G/DL
AST	66	20-65 U/L

Given the prior presentations of chromosome 13q deletion syndrome, an echocardiogram and renal ultrasound were performed, which were found to be normal.

Treatment

The patient required supplemental oxygen via nasal cannula due to experiencing episodes of desaturation. A follow-up CBC showed a normal platelet count; the feeding improved, and the patient began to gain weight. Genetic counseling and parental chromosome analysis were recommended to rule out the possibility of a balanced rearrangement. Upon discharge, the patient was weaned off of supplemental oxygen and was breathing room air without difficulty. He was breastfeeding well, gaining weight, and rooming in with his parents. The patient had reached a stable condition and was ready to be discharged home.

Follow-up

After being released from the hospital, the patient developed significant difficulty swallowing, which has led to limited calorie intake. At subsequent appointments, the patient was found to have consistently fallen below the first percentile for weight and is at risk of malnutrition. Consequent laboratory values, including CBC and basic metabolic panel (BMP), have been found to be within the normal range. At the patient's six-month follow-up appointment with the geneticist, it was determined that his genetic abnormality and lack of caloric intake would result in stunted growth. To address this, he will receive enteral feeds through a nasogastric (NG) tube and will be regularly monitored by his primary care physician (PCP) for complications. The patient will also follow up with a pediatric urologist to address the adherence of the penis to the scrotal sac. Complications will be addressed as they present during the patient's development as we are uncertain as to what will be the definitive effects of his chromosomal deletion.

## Discussion

Point mutations and karyotypic changes represent two types of potential chromosomal modifications. The phenotypic variations resulting from the intricate mechanisms governing gene expression can be astounding. Although investigating such variations can be challenging, it is crucial to recognize that cases involving chromosome or gene mutations serve as "natural experiments" and have contributed to the identification of genetic causes underlying several common congenital anomalies [10]. The occurrence and severity of genitourinary and anorectal abnormalities in individuals with 13q deletion syndrome are diverse and atypical. While some male patients present with hypospadias (distal) but no anorectal abnormalities, others exhibit severe hypospadias, anal atresia, and perineal issues. In this report, we discuss a case of ambiguous genitalia resulting from chromosome 13q deletion syndrome.

Research on human embryos has revealed that the anorectal canal and urogenital sinus develop from the primitive cloaca. A cascade of molecular processes regulates cloacal septation, scrotal closure, perineal closure, urethral tubularization, and penoscrotal placement [11]. In our case, a male child was diagnosed with chromosome 13q deletion syndrome accompanied by ambiguous genitalia, a rare medical condition. It is possible that our patient exhibits EFNB2 haploinsufficiency, which could explain his urogenital abnormalities [2]. Additionally, Wang et al. (2017) [5] have proposed that the location of the EDNRB gene on chromosome 13q22.3 might also contribute to urogenital abnormalities.

Studies have indicated that the EFNB2 gene, located in 13q33.3, which is not currently recognized as disease-causing by the Online Mendelian Inheritance in Man (OMIM), could be a promising candidate gene for conditions associated with 13q deletion syndrome, including hypospadias and anorectal issues [7,12,8]. However, Brown et al. (1993) [12] have observed that deletions affecting band 13q32 are often associated with a range of severe deformities, whereas deletions in the proximal region (q13-q31) are linked to mental impairment without significant abnormalities except for retinoblastoma. They found that deletions in q33-qter were associated with moderate-to-severe mental impairment, but genitourinary/anorectal malformations were not specifically identified as significant abnormalities. Unfortunately, deciphering the banding patterns of deleted chromosomes presents challenges in terms of karyotype/phenotype associations. Our patient with 13q deletion syndrome exhibited XY genitalia ambiguity, which has been reported in other case studies as well [13,14].

This research primarily focuses on male urogenital/anorectal health as a consequence of chromosome 13q deletion syndrome. Hypospadias, a common medical condition, can range from moderate to severe, with only 15% of cases being proximal. Penoscrotal transposition and anorectal abnormalities have been associated with proximal hypospadias. According to various studies, between 2.2% and 10.5% of individuals with hypospadias also present anorectal abnormalities [15].

## Conclusions

Further advancements in understanding chromosome 13q deletions can be achieved by establishing stronger correlations between karyotype and phenotype, as well as identifying unique phenotypes associated with the haploinsufficiency of specific genes. Additionally, maintaining a detailed record of the patient's development will be necessary to further document the long-term effects of chromosome 13q deletion, enabling us to provide better care for patients with this chromosomal deletion.
